# Supplementary material for: Polycyclic Aromatic Hydrocarbons Detected in Processed Meats Cause Genetic Changes in Colorectal Cancers
Source: Int J Mol Sci. 2021 Oct 11;22(20):10959. doi: 10.3390/ijms222010959 (PMC8537007; doi:10.3390/ijms222010959)
Supplement: Supplementary file 1 [file ijms-22-10959-s001.zip › Table S1.pdf]

Table S1. Average levels of PAHs detected in supermarket meat samples in ug/kg. (nd = not detected).

	ACNY	FL	PHEN	ANTH	PYR	B[a]A	CHRY	B[b]F	B[k]F	B[a]P	I[123-cd]P	D[a]A	B[ghi]P	Sum PAHs
Roast turkey	nd	1.78	14.29	16.03	1.18	nd	nd	28.92	28.55	nd	nd	nd	nd	90.75
Roast beef	nd	1.22	10.96	12.71	0.29	nd	nd	2.32	3.98	13.50	nd	nd	nd	44.97
Roast chicken	nd	1.34	11.53	13.98	0.41	nd	nd	nd	0.58	2.29	nd	nd	nd	30.13
Frankfurt	nd	1.04	12.56	15.04	nd	nd	nd	nd	2.60	1.21	nd	nd	nd	32.44
Single smoked ham	nd	nd	11.13	12.92	nd	nd	nd	nd	nd	nd	nd	nd	nd	24.04
Double smoked ham	nd	1.20	18.30	21.08	nd	nd	0.73	nd	nd	nd	nd	nd	nd	41.31
Triple smoked ham	nd	2.37	23.32	27.02	nd	nd	nd	nd	nd	nd	nd	nd	nd	52.72
Salami	1.84	11.14	28.68	34.13	nd	nd	nd	nd	nd	nd	nd	nd	nd	75.80
Sum PAHs	1.84	20.09	130.77	152.90	1.87	-	0.73	31.23	35.71	17.00	-	-	-	
Mean [PAH]	1.84	5.02	29.06	33.98	0.94	-	0.73	20.82	14.28	8.50	-	-	-	
SD	0.00	6.97	38.63	45.19	0.74	-	0.00	16.07	16.53	7.94	-	-	-	
Frequency detected	12.5%	87.5%	100.0%	100.0%	37.5%	-	12.5%	25.0%	37.5%	37.5%	-	-	-	
