# Supplementary material for: Polycyclic Aromatic Hydrocarbons Detected in Processed Meats Cause Genetic Changes in Colorectal Cancers
Source: Int J Mol Sci. 2021 Oct 11;22(20):10959. doi: 10.3390/ijms222010959 (PMC8537007; doi:10.3390/ijms222010959)
Supplement: Supplementary file 1 [file ijms-22-10959-s001.zip › Table S2.pdf]

Table S2. Forward and reverse primer sequences used for qPCR.

Target gene	Forward primer	Reverse primer
GAPDH	5’–CTTTTGCGTCGCCAG	5’–TTGATGGCAACAATATCCAC
TP53	5’–ACCTATGGAAACTACTTCCTG	5’-ACCATTGTTCAATATCGTCC
APC	5’–AGAGGTCATCTCAGAACAAG	5’-CATGTTGATTTCTCCCACTC
CTNNB1	5’–CTTGGAATGAGACTGCTG	5’-AGAGTGAAAAGAACGATAGC
KRAS	5’–GGCCTGCTGAAAATGACTG	5’-CTTGCTTCCTGTAGGAATCCTC
