# Supplementary figures and images for: Polycyclic Aromatic Hydrocarbons Detected in Processed Meats Cause Genetic Changes in Colorectal Cancers
Source: Int J Mol Sci. 2021 Oct 11;22(20):10959. doi: 10.3390/ijms222010959 (PMC8537007; doi:10.3390/ijms222010959)

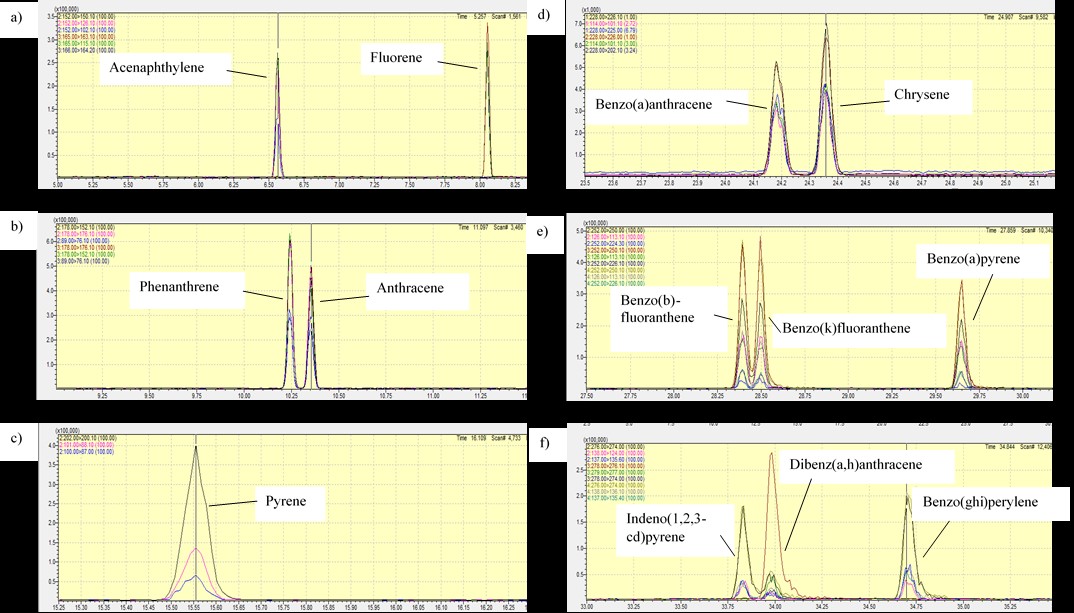

Supplement: Supplementary file 1 [file ijms-22-10959-s001.zip › Figure S1.jpg]

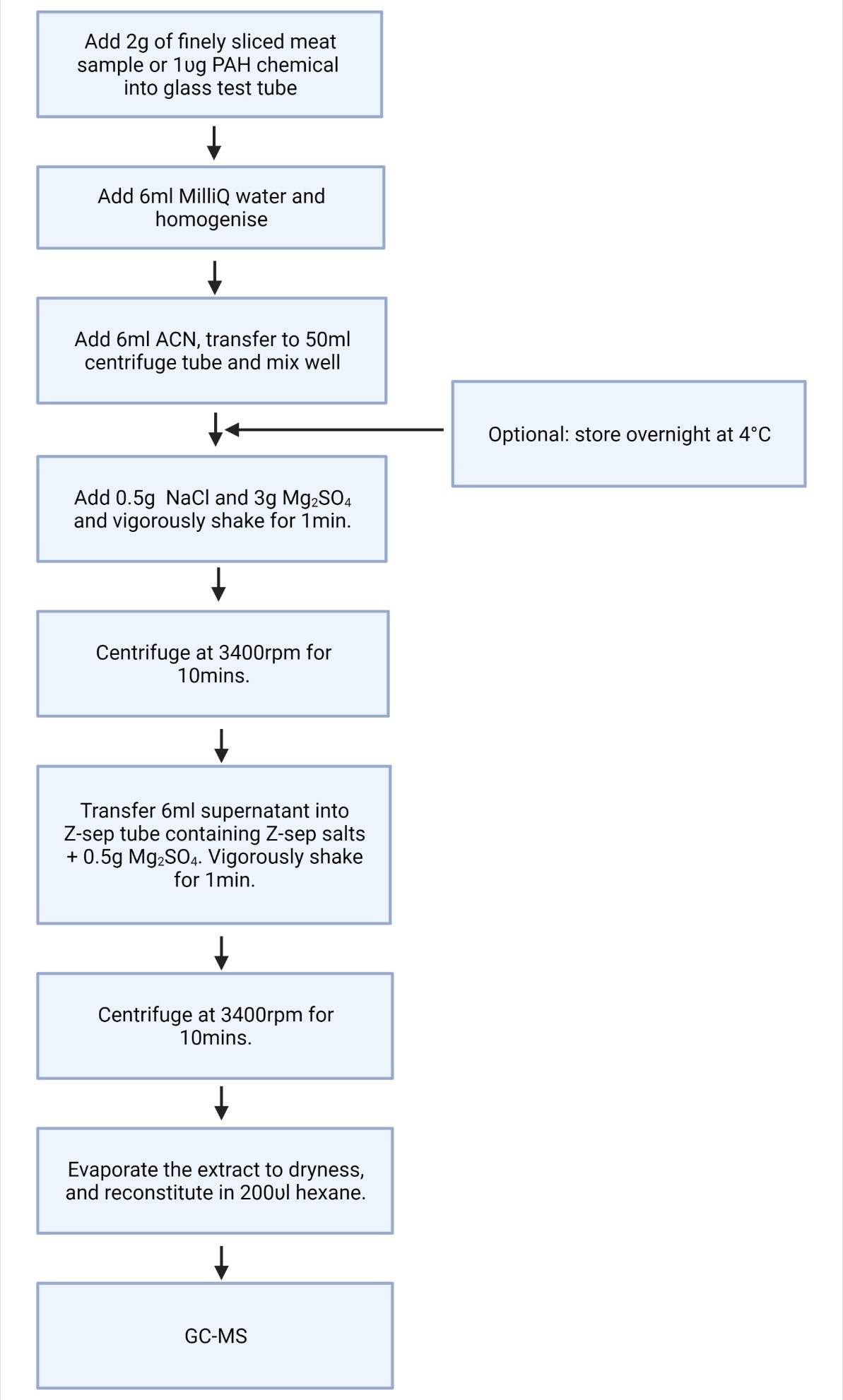

Supplement: Supplementary file 1 [file ijms-22-10959-s001.zip › Figure S2.jpg]
